# A Cyclodextrin‐Hosted Ir(III) Complex for Ratiometric Mapping of Tumor Hypoxia In Vivo

**DOI:** 10.1002/advs.202004044

**Published:** 2021-02-05

**Authors:** Peng Xiao, Chunyan Liu, Tiancong Ma, Xiuhong Lu, Lihong Jing, Yi Hou, Peisen Zhang, Gang Huang, Mingyuan Gao

**Affiliations:** ^1^ Key Laboratory of Colloid, Interface and Chemical Thermodynamics Institute of Chemistry Chinese Academy of Sciences Beijing 100190 P. R. China; ^2^ School of Chemistry and Chemical Engineering University of Chinese Academy of Sciences Beijing 100049 P. R. China; ^3^ Shanghai Key Laboratory of Molecular Imaging Shanghai University of Medicine and Health Sciences Shanghai 201318 P. R. China

**Keywords:** cyclodextrin, Ir(III) complex, oxygen level in vivo, quantitative mapping, tumor hypoxia

## Abstract

Hypoxia is considered as a key microenvironmental feature of solid tumors. Luminescent transition metal complexes particularly those based on iridium and ruthenium have shown remarkable potentials for constructing sensitive oxygen‐sensing probes due to their unique oxygen quenching pathway. However, the low aqueous solubility of these complexes largely retards their sensing applications in biological media. Moreover, it remains difficult so far to use the existing complexes typically possessing only one luminescent domain to quantitatively detect the intratumoral hypoxia degree. Herein, an Ir(III) complex showing red emissions is designed and synthesized, and innovatively encapsulated within a hydrophobic pocket of Cyanine7‐modified cyclodextrin. The Ir(III) complex enables the oxygen detection, while the cyclodextrin is used not only for improving the water solubility and suppressing the luminescence quenching effect of the surrounding aqueous media, but also for carrying Cyanine7 to establish a ratiometric oxygen fluorescence probe. 2D nuclear magnetic resonance is carried out to confirm the host–guest structure. The oxygen‐responsive ability of the resulting ratiometric probe is evaluated through in vitro cell and multicellular experiments. Further animal studies about tumor oxygen level mapping demonstrate that the probe can be successfully used for quantitatively visualizing tumor hypoxia in vivo.

## Graphical abstract

Cyanine7labeled *β*‐cyclodextrin is used to encapsulate an hydrophobic oxygen sensitive Iridium complex to form a sensitive ratiometric fluorescence probe that can be used for quantitatively detecting the hypoxia degrees of both cytoplasm and nucleus of cancer cells in vitro. Owing to the outstanding sensitivity, it can also be used for quantitatively mapping the oxygen levels of tumors in vivo.

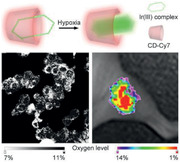

## 1 Introduction

The uncontrollable proliferation of tumor cells occurs typically faster than that of normal cells, leading to rapid consumption of oxygen released from surrounding vasculatures.^[^ ^1^, ^2^ ^]^ In consequence, hypoxia is prone to be induced and becomes an important signature of the majority of solid tumors.^[^ ^3^, ^4^ ^]^ The anoxic atmosphere can activate hypoxia‐inducible transcription factor 1 (HIF‐1) to initiate the expression of hypoxia‐responsive genes, which allows the tumor cells to survive and even proliferate under hypoxic conditions.^[^ ^5^, ^6^ ^]^ In addition, tumor hypoxia can vary the expression level of regulatory transcription factors and therefore plays a very important role in tumor progression and resistance to chemotherapy and radiotherapy.^[^ ^7^, ^8^, ^9^ ^]^ In this context, to develop precise oxygen detection methods for monitoring the tumor microenvironmental oxygen level in vivo is meaningful for understanding the malignant behaviors of tumors in association with therapies, prognosis, etc.^[^ ^10^ ^]^

Many early efforts have been devoted to detecting tumor hypoxia indirectly through bioredox chemistry methods.^[^ ^11^ ^]^ For example, nitroimidazole and azobenzene derivatives are usually chosen for constructing probes for oxygen sensing,^[^ ^12^ ^]^ because the up‐regulated reductases such as nitroreductase and azoreductase in hypoxia environment will reduce the corresponding nitro and azo groups that quench the fluorescence of fluorophore moieties in their native state. Relaying on such hypoxia‐assisted off‐on switch, the hypoxia of tumor microenvironment is detected. However, it is nearly impossible to monitor the real‐time oxygenation level as a significant period of time is required for reductases to activate the fluorescence of nitroimidazole and azobenzene probes, especially in environment with lowered pH.^[^ ^13^ ^]^

Apart from the aforementioned indirect detections, direct detections of oxygen level based on the energy transfer between oxygen and dye in excited triplet state have also been reported.^[^ ^14^, ^15^ ^]^ Typically, these probes are chosen from transition metal ruthenium(II) and iridium(III) complexes whose fluorescence can be quenched to some extent, depending on the oxygen concentration.^[^ ^16^, ^17^, ^18^ ^]^ For example, red phosphorescent iridium(III) complexes were reported for monitoring the intracellular O_2_ content in vitro through confocal luminescence imaging.^[^ ^19^ ^]^ Nevertheless, such turn‐off type of probes is not suitable for quantitatively detecting the oxygen levels in vivo because the fluorescence signal is dependent not only on the oxygen level but also on the local probe concentration. To address this issue, oxygen‐insensitive fluorophores including organic dyes, quantum dots (QDs), and upconversion nanoparticles were chosen as internal reference for building up versatile ratiometric fluorescent probes. For example, a nanoprobe composed of QDs, Ir(III) complex, and glycerol monoolein was fabricated for quantitative hypoxia sensing in vitro.^[^ ^20^ ^]^ Nevertheless, the intensity ratio between the fluorescence of QDs and Ir(III) was only varied by a factor of 2.8 when the oxygen content was tuned from 21% to 1%, a relevant oxygen range for biological tissues. Therefore, there remains a large room to further improve the sensitivity of oxygen sensing.

Fluorescence quantum yield (QY) is apparently a major parameter for improving the sensing sensitivity. In this respect, multiheterocycle ligands are proposed for improving the fluorescence QY of transition metal complexes, but they inevitably increase the hydrophobicity of the resulting probes, which is unfavorable for direct oxygen sensing. By inserting the hydrophobic Ir(III) complexes into mesoporous silica, or covalently attaching them on the surface of poly(N‐vinylpyrrolidone)‐modified nanoparticles, these complexes could be transferred into aqueous systems.^[^ ^21^ ^]^ Unfortunately, the fluorescence QY was dramatically quenched by newly formed non‐radiative recombination channels induced by strong polar water molecules surrounding the Ir(III) complexes. Until now, it remains very much challenging to use multiheterocycle ligands to construct highly fluorescent oxygen probe used for aqueous systems.

Herein, a ratiometric O_2_ probe was constructed by using *β*‐cyclodextrin (*β*‐CD) to host a hydrophobic red‐emitting complex formed by iridium with two cyclometalated ligands and one bidentate ancillary ligand, i.e., bis(2‐(2’‐benzothienyl)‐pyridinato‐N,C^3’^) iridium 4,6‐dioxoheptanoic acid (denoted as Ir‐BTPHSA). With a hydrophobic interior and hydrophilic exterior, CDs form complexes with hydrophobic compounds and thus are widely used for delivering a variety of hydrophobic drugs.^[^ ^22^, ^23^ ^]^ In the current studies, the *β*‐CD molecules were labeled with oxygen‐insensitive fluorescent Cyanine7 (Cy7) to achieve ratiometric fluorescence together with the encapsulated Ir(III) dye for quantitative oxygen sensing. 2D nuclear magnetic resonance (2D NMR) was further carried out to analyze the host–guest interactions between *β*‐CD and Ir‐BTPHSA dye. The ratiometric oxygen‐responsiveness of the as‐prepared probe was confirmed through in vitro cell experiments, followed by tumor imaging studies to demonstrate the capacity of the current probe for quantitative detection of oxygen levels in vivo.

## 2 Results and Discussion

### 2.1 Preparation and Characterization of Ir‐BTPHSA

**Figure 1:**
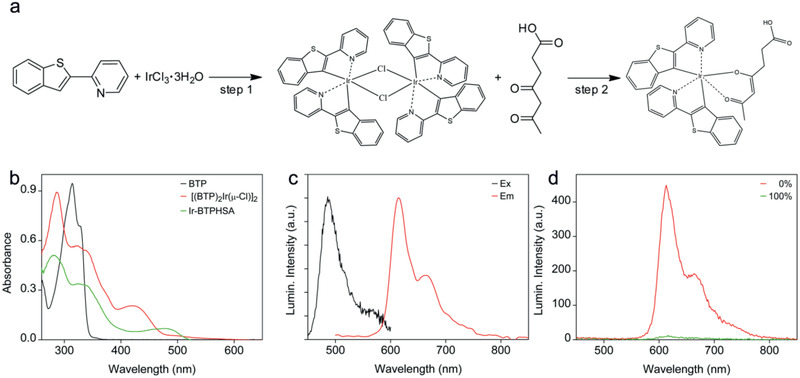
a) Synthetic procedures for Ir‐BTPHSA, b) UV–vis absorption spectra of BTP, [(BTP)_2_Ir(*μ*‐Cl)]_2_, and Ir‐BTPHSA, c) excitation and emission spectra of Ir‐BTPHSA, d) emission spectra of Ir‐BTPHSA recorded under 0% and 100% oxygen level, respectively.

The Ir‐BTPHSA complex is formed by iridium(III) together with two cyclometalated ligands and one bidentate ancillary ligand. Its synthetic procedures are shown in **Figure** **1**a. As aforementioned, multiheterocycle ligands are preferred for forming complexes with improved fluorescence QY. Nevertheless, using C^N ligand to construct six‐coordination complex of Ir(III) will inevitably increase the probability of water molecules to attack Ir(III), leading to the collapse of the complex in low pH environment.^[^ ^24^, ^25^ ^]^ Compared to N‐, C‐, or P‐donor ligands, O‐donor ligand especially diketonate‐like ligand can effectively improve the stability and the photoluminescence (PL) efficiency as well.^[^ ^26^ ^]^ Therefore, 4,6‐dioxoheptanoic acid was chosen as the ancillary ligand in the current work. In addition, the carboxylic moiety of 4,6‐dioxoheptanoic acid was expected for further functionalization of the resulting complex. More importantly, to maintain a suitable size for forming host–guest complex with CDs was also taken into consideration when designing the complex.

The formation of the metal complexes was followed by both absorption and fluorescence spectroscopies at different stages. As shown in Figure 1b, the coordination between Ir(III) and BTP/4,6‐dioxoheptanoic acid leads to dramatic changes in absorption. For example, the major absorption peak of BTP initially located at 314 nm disappears after forming the chloride‐bridged dimer, i.e., [(BTP)_2_Ir(*μ*‐Cl)]_2_. Instead, three new absorption bands appear at 285, 322, and 420 nm, respectively. The former two bands can be assigned to *π*–π* transitions,^[^ ^26^ ^]^ while the latter one can be assigned to metal‐to‐ligand charge‐transfer ^1^MLCT.^[^ ^27^ ^]^ After the following reaction between [(BTP)_2_Ir(*μ*‐Cl)]_2_ and 4,6‐dioxoheptanoic acid, the absorption band at 420 nm shifts to 480 nm. Different from chloride, O‐donor ancillary ligand will modulate the electron density of Ir(III) by the interplay between *σ*‐withdrawing and *π*‐donating properties at the metal center, thus enhance both spin‐orbit coupling ^3^ *π*–π*, and ^3^MLCT transitions.^[^ ^28^ ^]^

The excitation and emission spectra of Ir‐BTPHSA are shown in Figure 1c. Under ambient conditions, the main emission peak of Ir‐BTPHSA is located at 615 nm with a shoulder at around 663 nm. The intensity of these emissions can remarkably be varied by oxygen levels. As shown in Figure 1d, the PL intensity of Ir‐TBPHSA in aqueous solution can be increased by a factor of ≈44 as the oxygen content is decreased from 100% to 0%. It is deserved to mention that 5 min is enough for oxygen to quench the fluorescence of Ir‐BTPHSA.

### 2.2 Hydrophilic Modification of Ir(III) Complex

**Figure 2:**
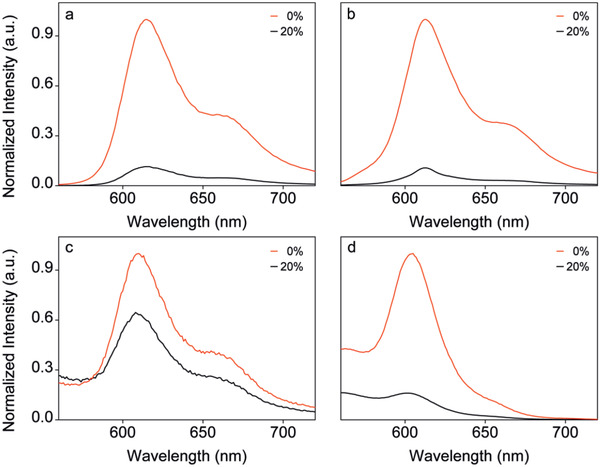
The PL spectra of Ir‐BTPHSA in different forms or different media recorded at oxygen levels of 0% and 20%, respectively (ex = 488 nm): a) Ir‐BTPHSA in DMSO, b) jeffamine‐modified Ir‐BTPHSA in DMSO, c) Ir‐BTPHSA loaded by PEGylated nanoparticles in aqueous solution, and d) Ir‐BTPHSA encapsulated by CDs (25 mg mL^−1^) in aqueous solution.

Although Ir‐BTPHSA presents very remarkable and quick responsiveness to oxygen, the poor aqueous solubility limits its biomedical applications.^[^ ^29^, ^30^ ^]^ To address this issue, two different strategies were proposed. One is to covalently attach Ir‐BTPHSA on the surface of PEGylated NaGdF_4_:Yb,Tm@NaGdF_4_ nanoparticles shown in Figure S1 in the Supporting Information via 2,2’‐(ethylenedioxy)bis(ethylamine) (jeffamine), the other is to encapsulate Ir‐BTPHSA with *β*‐CD. The results on the oxygen‐dependent fluorescence shown in **Figure** **2**a,b reveal that jeffamine modification doesn't vary the sensitivity of Ir‐BTPHSA to oxygen, showing an on–off (0% vs 20% oxygen) ratio around 9.1. However, the sensitivity is remarkably reduced after Ir‐BTPSHA carried by the PEGyglated nanoparticles is transferred into aqueous system (Figure 2c). Fortunately, the sensitivity remains high in aqueous solution if Ir‐BTPSHA is encapsulated by *β*‐CD, with on–off close to 6.2 (Figure 2d). The detailed absorption spectra of all samples mentioned in Figure 2 are provided in Figure S2 in the Supporting Information.

**Figure 3:**
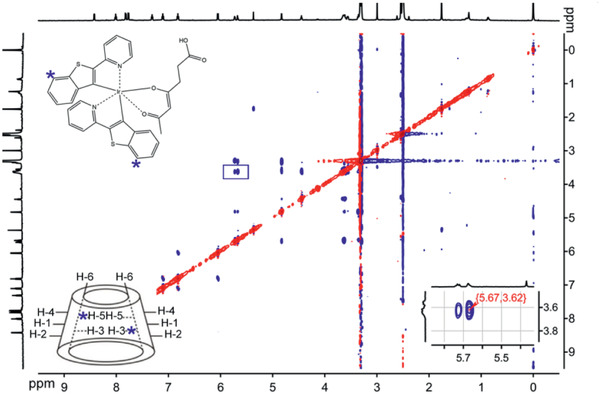
2D‐ROESY spectra of Ir‐BTPHSA/CD‐NH_2_ (Note: H—C^4’^ and H—C^7’^ atom of Ir‐BTPHSA are indicated by blue stars, while the erea highlighted by blue square is enlarged in the right bottom corner to show the details of the region of interest).

In fact, CDs are widely used for loading hydrophobic drugs, but they were never used to our best knowledge for encapsulating hydrophobic dyes for oxygen sensing. The result shown in Figure 2d suggest that Ir‐BTPHSA probably hides in the hydrophobic cavity of *β*‐CD to avoid direct contact with water. To verify this hypothesis, 2D NMR spectroscopy including 2D rotating‐frame Overhauser effect spectroscopy (2D‐ROESY) was adopted to study the binding geometries of Ir‐BTPHSA with *β*‐CD. As the cross peaks in the ROESY spectra can be used to identify nuclei that are closely positioned in space,^[^ ^31^, ^32^ ^]^ 2D ROESY was used in the current study to analyze the spatial proximity of the host–guest complexation between *β*‐CD and Ir‐BTPHSA. As shown in **Figure** **3**, the major cross peaks are located at 3.62 and 5.67 ppm, respectively. The signal peaking at 3.62 ppm can be assigned to H‐3 (3.61 ppm) and H‐5 (3.57 ppm) of *β*‐CD according to literature,^[^ ^23^ ^]^ while the signal at 5.67 ppm can be assigned to H—C4’ or H—C7’ of Ir‐BTPHSA.^[^ ^27^ ^]^ Therefore, it can be concluded that Ir‐BTPHSA is encapsulated in the hydrophobic cavity of *β*‐CD with the most probable binding geometry shown in Figure S3 in the Supporting Information. The formation of such complex structure dramatically increased the solubility of Ir‐BTPHSA in aqueous system as shown in Figure S4 in the Supporting Information. In order to show the excellent stability of the CD‐encapsulated Ir‐BTPHSA probe in bioenvironments, particularly tumor microenvironment, several simulative media containing different concentrations of H_2_O_2_, GSH, or hydrogen ion were adopted to exam the stability of the resulting probe through fluorescence as it is very sensitive to the complex structure. The results given in Figure S5 in the Supporting Information suggested that the Ir‐BTPHSA encapsulated by *β*‐CD is very stable in environment featured by typical abnormal factors.

### 2.3 Preparation and Characterization of the Ratiometric Oxygen Probe

**Figure 4:**
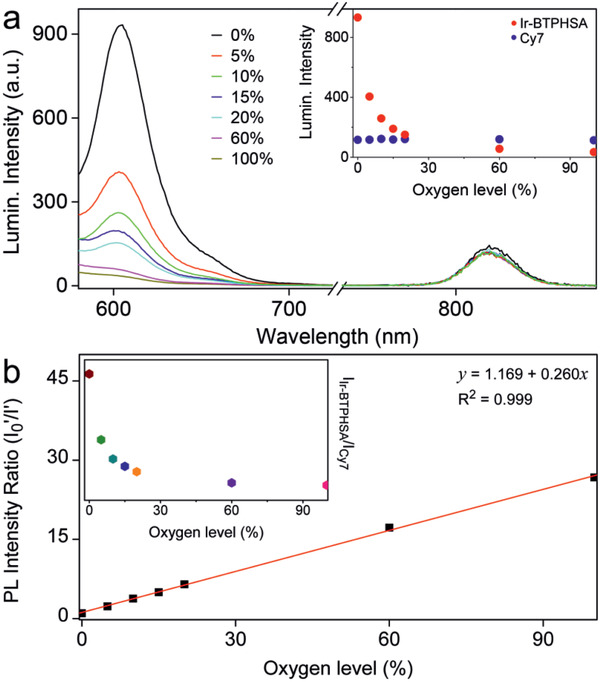
a) Luminescence spectra of Ir‐BTPHSA/CD‐Cy7 recorded under various oxygen levels upon excitation at 488 and 747 nm for Ir‐BTPHSA complex and Cy7, respectively (Inset: PL intensities of Ir‐BTPHSA and Cy7 against oxygen levels), b) a liner fitting of *I*_0_^′^/*I*′ against the oxygen level (*I*_0_^′^ and *I*′ refer to PL intensities of Ir‐BTPHSA recorded at 0% oxygen and specific oxygen levels, respectively, after normalized with reference PL intensity of Cy7) (inset: normalized PL intensity of Ir‐BTPHSA against the oxygen level).

In order to quantitatively detect the oxygen level through imaging, Cy7 was used to covalently label *β*‐CD to form a ratiometric fluorescence probe together with Ir‐BTPHSA inside *β*‐CD. The photoluminescence (PL) of Cy7‐labeled Ir‐BTPHSA/CD probe (denoted as Ir‐BTPHSA/CD‐Cy7) was recorded against the oxygen level. As shown in **Figure** **4**a, Ir‐BTPHSA/CD‐Cy7 presents a very oxygen‐sensitive fluorescence at 606 nm, while the fluorescence of Cy7 at 819 nm is almost independent of the oxygen level.

Oxygen sensing with luminescent molecules (usually phosphorescent molecules) is usually based on bimolecular quenching process of the excited molecules with molecular oxygen. An energy diagram describing oxygen quenching of the excited triplet state of a dye together with the rate constants of the dominant relaxation processes was described in literature.^[^ ^33^ ^]^ In general, oxygen quenching involves two competing pathways for the lowest excited triplet state of the oxygen probe, i.e., non‐charge transfer and charge transfer. According to literature,^[^ ^33^ ^]^ the latter is the dominant process for Ir(III) complexes. Thus, the kinetics of oxygen‐induced quenching follows the Stern–Volmer equation (Equation (1)) that describes the relationship between lifetime (or quantum yield) and oxygen pressure (or concentration)

(1) $$\begin{array}{cc} \frac{\emptyset_{p}^{0}}{\emptyset_{p}} & =\frac{\tau_{p}^{0}}{\tau_{p}}=1+k_{q}\tau_{p}^{0}\;p_{O_{2}}=1+k_{\mathrm{sv}}\;p_{O_{2}}\\ & =1+k_{q}^{\prime }\tau_{p}^{0}\;\left[O_{2}\right]=1+k_{\mathrm{sv}}^{\prime }\left[O_{2}\right] \end{array}$$

where $\emptyset_{p}^{0}$ and $\tau_{p}^{0}$ are quantum yield and lifetime under 0% oxygen concentration, respectively, ∅_p_ and *τ*_p_ are the corresponding values determined under a specific oxygen pressure of $p_{O_{2}}$ or oxygen concentration of [O_2_], and *k*_q_ (or *k*_q_′) and *k*_sv_ (or *k*_sv_′) are bimolecular quenching rate constants and Stern–Volmer constants, respectively.^[^ ^33^ ^]^ To more accurately correlate the PL intensity of Ir‐BTPHSA with oxygen concentration, as shown in Figure 4a, the PL intensity of Ir‐BTPHSA is normalized with respect to Cy7 to exclude any possible concentration effect. Then, the ratios of the PL intensities of Ir‐BTPHSA to Cy7 (denoted as *I*_Ir‐BTPHSA_/*I*_Cy7_), equal to $\emptyset_{p}^{0}/\emptyset_{p}$, were recorded under 0% oxygen (denoted as *I*_0_′) and a specific oxygen level (denoted as *I*′) respectively, and then plotted against the oxygen level. The perfect linear relationship between *I*_0_′/*I*′ and oxygen level shown in Figure 4b clearly demonstrates that the responsiveness of the current Ir‐BTPHSA/CD‐Cy7 probe perfectly follows the Stern–Volmer equation. As the absorption of Ir‐BTPHSA is independent of the oxygen levels as shown in Figure S6 in the Supporting Information, it can be concluded that a dynamic quenching process is responsible for the oxygen sensing of the current probe. It merits a specific mention that the normalized PL intensity of Ir‐BTPHSA loaded by *β*‐CD is decreased by a factor of 27.1 when oxygen level was increased from 0% to 100%, showing an outstanding sensitivity for oxygen sensing.

### 2.4 Cellular hypoxia imaging in vitro

**Figure 5:**
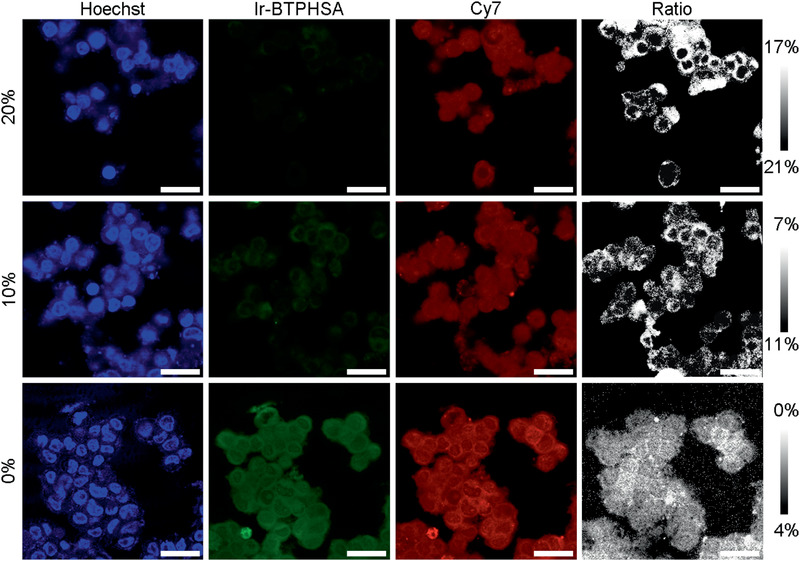
Confocal luminescence images of living LS180 cells stained with Ir‐BTPHSA/CD‐Cy7 and Hoechst dyes captured after incubation of 15 min at oxygen levels of 0%, 10%, and 20%, respectively. The last column of images were obtained for showing the hypoxia situations within the tumor cells after being incubated at different oxygen levels. The scale bar is 25 µm.

The ratiometric probe was then used to examine the cellular oxygen levels in LS180 cells that were incubated with the probe under the oxygen concentrations of 0%, 10%, and 20%, respectively. Previous studies suggest that cancer cells are apt to be enriched with more lipophilic cations than normal cells due to their more negative potentials. Therefore, there is a salient affinity between organelles (including mitochondria, nucleus) and cyclometalated Ir(III) complexes,^[^ ^34^ ^]^ which explains the staining results on cancer cells shown in **Figure** **5**. It is very apparent that with the decrease of oxygen level, the luminescence intensity of Ir‐BTPHSA is remarkably increased. Specifically, in comparison with that recorded under 20% oxygen, the PL intensity of Ir‐BTPHSA determined in the anoxia atmosphere is enhanced by a factor of 5, while the stained cells also exhibit much less unaffected but generally increased red emissions through Cy7 channel. According to the results given in Figure 4a, the emission of Cy7 should be independent of oxygen level, the slightly increased PL intensity of Cy7 against the decrease of oxygen level indicates that the oxygen level may affect the ability of the cancer cells in uptaking foreign probes. Fortunately, Cy7 as internal reference can well exclude the interference of probe concentration, which is particularly important for quantitative detection of oxygen level in vivo because the concentration and localization of the probes can be very different within the body. It is also deserved to mention that apart from cytoplasm the cell nuclei also present strong signals from both Ir‐BTPHSA and Cy7, which offers a possibility to detect and even compare the nucleus hypoxia with the hypoxic situation in cytoplasm in vitro. To our best knowledge, there is no hypoxia probe capable of doing so. By taking this advantage, 2D hypoxia mapping of the whole cells was obtained according to the linear relationship between *I*_0_′/*I*′ and oxygen level shown in Figure 4b. According to the hypoxia mapping results given in the last column in Figure 5, the cytoplasm has apparently lower oxygen level than the cell nucleus according to the hypoxia signal defined by *I*_Ir‐BTPHSA_/*I*_Cy7_. But this difference gets smaller and smaller when the environmental oxygen level is decreased from 20% to 0%, which was never observed before.

**Figure 6:**
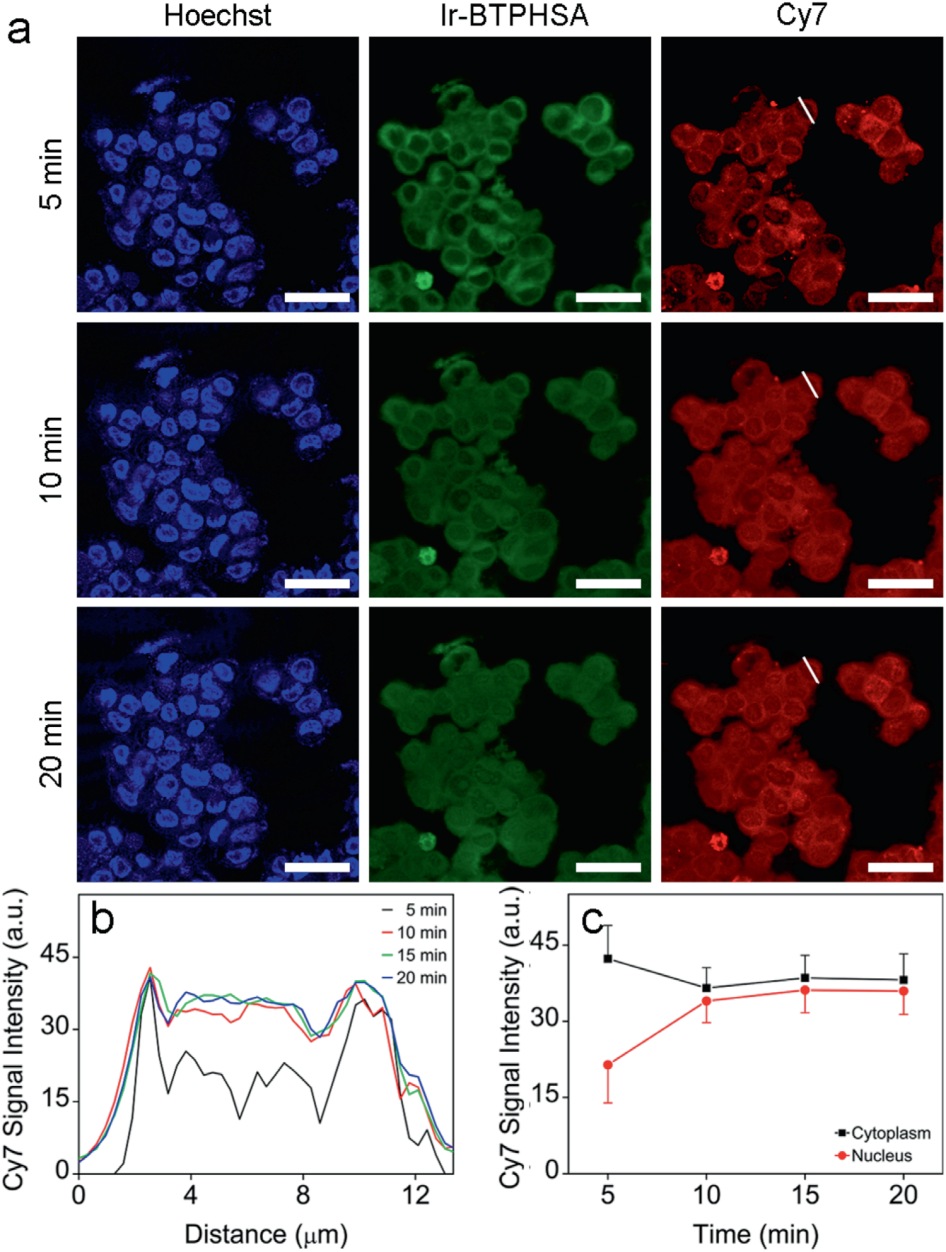
a) Time‐elapse confocal luminescence images of living LS180 cells recorded at different time points upon incubation with Ir‐BTPHSA/CD‐Cy7, b) Cy7 luminescence intensity profiles across a selected cell indicated by white short lines recorded at different time points, c) the average Cy7 signals extracted from cytoplasm and nucleus of cells incubated with Ir‐BTPHSA/CD‐Cy7 for different periods of time.

A series of experiments were also performed to follow the dynamic signal variations at different cell locations through in situ time‐elapse imaging (**Figure** **6**a). According to the line‐scanning results shown in Figure 6b, 5 min is enough for CD‐encapsulated Ir‐BTPHSA probes to show stable cytoplasm signals from both Ir‐BTPHSA and Cy7 in cells incubated in oxygen‐free environment, whereas the signals from cell nuclei keep increasing until 10 min to become stabilized according to the statistical results shown in Figure 6c. Thus, the current probes can be used for almost real‐time monitoring of hypoxia in vivo, owing to their quick uptake by cancer cells. Normally, the imaging probes can be internalized into cells through four different pathways including receptor‐mediated endocytosis, caveolae, pinocytosis, and phagocytosis. As the complex Ir‐BTPHSA are encapsulated by *β*‐CD, it can be reasonably expected that the current Ir‐BTPHSA/CD‐Cy7 probes are internalized into cells via pinocytosis according to literature studies on the cellular uptake of CD.^[^ ^35^ ^]^ Regarding the mechanism of the internalization into cell nuclei, the current probe is different from most of cyclometalated iridium (iii) complexes that show a tendency to be localized in mitochondria,^[^ ^36^ ^]^ which is probably because the ancillary ligand used in the current probe can influence the electron delocalization and thus enable the penetration of iridium(iii) complex across the nuclear membrane,^[^ ^37^ ^]^ supported by the facts that cyclodextrin‐complexed oligonucleotide presented an enhanced nuclear uptake in comparison with the uncomplexed oligonucleotide.^[^ ^38^ ^]^ According results given in Figure S7 in the Supporting Information, the signal overlap of these two dyes is around 97.2% in cells and the Pearson's coefficient for characterizing their colocalization is as high as 94.0%. The extremely high signal overlap suggests that the Ir‐BTPHSA/CD‐Cy7 probes are very stable in vitro. As an ideal imaging probe, it should be non‐ or low‐toxic to cells. Therefore, the cytotoxicity of the resulting probe was evaluated through MTT cell proliferation assay on colorectal cancer cell line LS180 cells. The results shown in Figure S8 in the Supporting Information revealed that the resulting probe had a very safe profile.

### 2.5 In Vivo Imaging of Tumor Hypoxia with the CD‐Modified Ratiometric Probe

**Figure 7:**
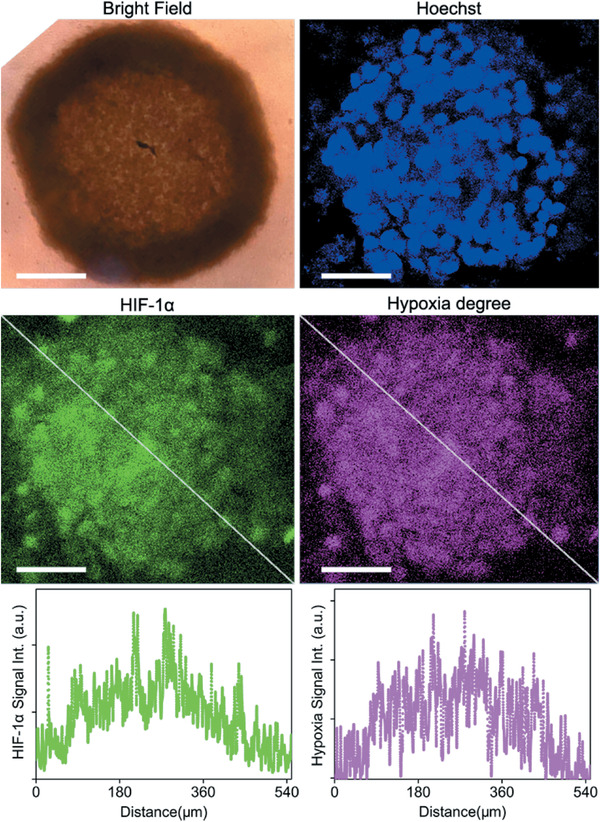
Confocal luminescence images of a representative LS180 multicellular spheriod co‐stained with Ir‐BTPHSA/CD‐Cy7 and FITC‐labeded antibody of HIF‐1*α*, together with the luminescence intensity profiles across the spheriod as indicated by the white dashed lines. The scale bars correspond to 100 µm.

All above results encouraged us to use the CD‐encapsulated Ir‐BTPHSA probes to monitor the tumor hypoxia in vivo. But before that, LS180 multicellular spheroids (MCs) were adopted to evaluate the current hypoxia probe as the intermediate complexity between in vivo tumors and in vitro monolayer cultures makes MCs suitable to reflect the heterogeneity of tumor microenvironment for probe screening and evaluation.^[^ ^39^ ^]^ For comparison, the same multicellular spheroids were also stained with FITC‐labeled antibody of HIF‐1*α* that can be viewed as a messenger sent to the nucleus to activate transcriptional responses to hypoxia.^[^ ^40^ ^]^ Therefore, the HIF‐1*α* level also reflects the hypoxia degree in cancer cells. According to the fluorescence image captured through FITC channel, the central area of a representative MC is apparently hypoxic due to the more compact packing structure of the cells, as shown in **Figure** **7**, contrasting to the traditional monolayer cell cultures.^[^ ^41^ ^]^ As this hypoxia characteristic is well be reflected by the signal ratio of *I*_Ir‐BTPHSHA_/*I*_Cy7_, it can be concluded that the ratiometric probe is sensitive enough to distinguish the hypoxic core from the normoxic surface.

**Figure 8:**
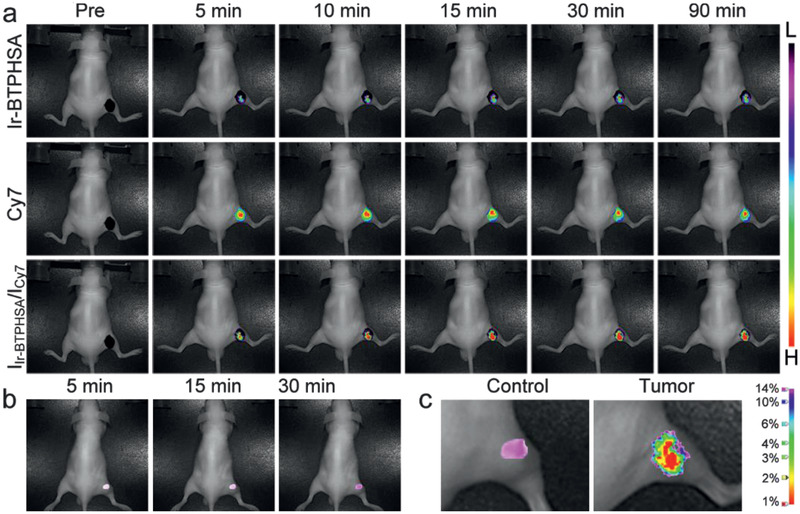
a) Photoluminescence images of the tumor site overlaid with the corresponding bright field images of tumor‐bearing mice captured at different time points postinjection of the ratiometric probe, b) photoluminescence images of a comparable site of mice bearing no tumor captured after subcutaneous injection of the same probe, c) oxygen level mapping of the tumor and its control site based on the normalized Ir‐BTPHSA signal and its correlation with oxygen level.

To show the oxygen‐responsiveness of the CD‐modified ratiometric probe in vivo, the mice bearing subcutaneously implanted tumor grown from colorectal cancer cell line LS180 were adopted and the ratiometric probe was intratumorally injected. The results given in **Figure** **8**a reveal that the probe can quickly respond to the hypoxia environment. Within 5 min, a detectable Ir‐BTPHSA signal is generated and reaches its intensity maximum within 30 min with reference to Cy7 signal. Thereafter, the hypoxia signal (*I*_Ir‐BTPHSHA_/*I*_Cy7_) remains nearly unchanged over the following 60 min. It is also deserved to mention that Ir‐BTPHSHA signals are mainly located in the central area of Cy7 signals, which is reasonably expected as the Ir‐BTPHSHA signal is sensitive to oxygen levels that are typically lower in the inner part of a tumor. According to control experiment that was carried out by injecting the hypoxia probe into similar region of mice bearing no tumor (Figure 8b), the average hypoxia signal is about 0.28. In order to quantitatively map the oxygen level within tumor, the Dalton's law of partial pressures stating that in a mixed gas each element exerts a pressure proportional to its fraction of the total volume (partial pressure)^[^ ^42^ ^]^ was introduced to correct the relationship between oxygen level (*x*%) and the partial pressure of oxygen in body

(2) $$x\;\%\;=\;\frac{p_{O_{2}}}{760-p_{0}}$$

where *x* represents oxygen level, $p_{O_{2}}$ is subcutaneous partial pressure of oxygen expressed in mmHg, and *p*_0_ is the saturated vapor pressure, i.e., 47.10 mmHg at 37 °C. And according the Equation (1), the relationship between *I*_0_′/*I*′ and $p_{O_{2}}\;$will be

(3) $$\frac{I_{0}^{\prime }}{I^{\prime }}=\;1.169+185.3\;\times p_{O_{2}}$$

The average oxygen partial pressure in the tissue is called the tissue partial pressure of oxygen, typically determined through methods including gasometry, electron paramagnetic resonance oximetry, and positron emission tomography, etc.^[^ ^43^ ^]^ The oxygen level for the subcutaneous non‐tumorous tissues is normally around 100 mmHg, corresponding to oxygen level of 14%.^[^ ^44^ ^]^ As this non‐invasively determined oxygen level falls in a very reasonable range determined through blood gas analysis,^[^ ^45^ ^]^ in the same way the oxygen level of hypoxia core of the tumor was then calculated according to the results given in Figure 8a. The resulting oxygen level of the very hypoxia core was around 7.1 mmHg (≈1% oxygen), well matching previous experimental results obtained by Eppendorf pO_2_ electrode system,^[^ ^46^ ^],^ e.g., less than 1% oxygen level for the hypoxia core of tumors larger than 9 mm. Although the current probes will diffuse throughout the entire tumorous region and even spread outside the tumor against time, the ratiometric signal can perfectly exclude this effect in mapping the hypoxia regions. In addition, the perfect linear relationship between the hypoxia signal variation and oxygen concentration shown in Figure 4 helps avoid the interference of emission light absorption and scattering by surrounding tissues.^[^ ^47^ ^]^ All these properties finally enable the quantitative mapping of oxygen level throughout the tumorous region, as given in Figure 8c, which can never be obtained through the Eppendorf pO_2_ electrode system apart from its invasive nature.

## 3 Conclusion

In summary, a simple and efficient strategy has been developed for obtaining biological oxygen sensor by encapsulating hydrophobic Ir(III) complex with *β*‐CD. By further labeling the CD host with Cy7, the ratiometric optical probe is successfully constructed for tumor hypoxia study. In vitro cell imaging results reveal that the obtained probe can quickly respond to O_2_ showing a remarkable sensitivity, while in vivo imaging experiments in combination with the data analysis demonstrate that the current probe can be used for non‐invasively mapping the hypoxia microenvironment of solid tumors and quantitatively determining the oxygen level in vivo.

## 4 Experimental Section

##### Chemicals

IrCl_3_·3H_2_O (99%, I111008), 2‐(2‐pyridyl) benzothiophene (97%, P121516), 2‐ethoxyethanol (>99%, E110822), *β*‐CD (98%, C104384) were purchased from Aladdin, 4,6‐dioxoheptanoic acid was purchased from Huateng Pharma Co., Ltd., 2,2’‐(ethylenedioxy)bis(ethylamine) and p‐toluene sulfonylchloride (p‐TsCl) were purchased from Sigma‐Aldrich, and Cy7‐NHS was purchased from GE healthcare. Other analytical grade chemicals, such as methanol, n‐hexane, diethyl ether, acetone, tetrahydrofuran (THF), and DMSO were purchased from Sinopharm Chemical Reagent Beijing, Co., Ltd. All chemicals mentioned above were used as received without any further purification.

##### Synthesis of Iridium (III) Complex (Ir‐BTPHSA)

The synthetic procedures for Ir‐BTPHSA are shown in Figure 1. In general, there were two steps involved. In the first step, IrCl_3_ reacted with 2‐(2‐pyridyl) benzothiophene (BTP) to form a cyclometalated Ir(III) *µ*‐chloride‐bridged dimer ([(BTP)_2_Ir(*μ*‐Cl)]_2_), and then coordinated with 4,6‐dioxoheptanoic acid (HSA) to form tris‐ligand complex Ir‐BTPHSA via a salt metathesis reaction. In detail, IrCl_3_·3H_2_O (0.1780 g, 0.5 mmol) and BTP (0.2114 g, 1.0 mmol) were dissolved in a mixture formed by 5 mL water and 15 mL 2‐ethoxyethanol, and the reaction mixture was heated to 125 °C and maintained at this temperature for 10 h. After being cooled to room temperature, the [(BTP)_2_Ir(*μ*‐Cl)]_2_ was precipitated and then collected through filtration, followed by thorough washing with water, methanol, and n‐hexane in sequence. ^1^H NMR (300 MHz, DMSO‐d_6_; *δ*, ppm): 9.93 (d, *J* = 5.5 Hz, 2H), 9.69 (d, *J* = 5.8 Hz, 2H), 8.15 (dt, *J* = 27.3, 7.9 Hz, 4H), 7.93 (d, *J* = 7.7 Hz, 2H), 7.79 (dt, *J* = 24.9, 12.4 Hz, 6H), 7.49 (dt, *J* = 22.3, 6.7 Hz, 4H), 7.16 (dt, *J* = 15.1, 7.3 Hz, 4H), 6.91 (t, *J* = 7.7 Hz, 2H), 6.78 (t, *J* = 7.5 Hz, 2H), 6.19 (d, *J* = 7.9 Hz, 2H), 5.55 (d, *J* = 8.2 Hz, 2H). The ^1^H NMR spectrum is shown in Figure S9 in the Supporting Information.

In the second step, [(BTP)_2_Ir(*μ*‐Cl)]_2_ (0.0672 g, 0.5 mmol) and 4,6‐dioxoheptanoic acid (0.0342 g, 2 mmol) were dissolved in 10 mL 2‐ethoxyethanol containing 0.145 mL trimethylamine. The reaction mixture was heated to 80 °C and maintained at this temperature for 15 h under nitrogen protection. After the reaction mixture was cooled down to room temperature, the resulting precipitates were collected by centrifugation, and then purified through preparative high performance liquid chromatography. MS (ESI, CH_3_CN + CH_2_Cl_2_): calcd. 874.272; found 874.04. ^1^H NMR (300 MHz, DMSO‐d_6_; *δ*, ppm): 12.04 (s, 1H), 8.46 (s, 1H), 7.80 (dd, *J* = 18.3, 6.7 Hz, 6H), 7.28 (dd, *J* = 16.4, 6.7 Hz, 2H), 7.11 (t, *J* = 7.6 Hz, 2H), 6.83 (t, *J* = 7.6 Hz, 3H), 6.08 (t, *J* = 8.7 Hz, 2H), 5.40 (s, 1H), 2.21 (m, 4H), 1.77 (s, 3H). The ^1^H NMR spectrum is shown in Figure S10 in the Supporting Information and the homonuclear chemical shift correlation spectroscopy (COSY) results are shown in Figure S11 in the Supporting Information. These results suggested that the resulting compound had a statisfying purity.

##### Synthesis of CD‐NH_2_

*β*‐CD (10.0 g, 0.0088 mol) was first dissolved in a mixture formed by 100 mL water and 70 mL THF containing KOH (0.4 g, 0.007 mol). Then, a p‐TsCl solution was prepared by dissolving p‐TsCl (1.7 g, 0.0088 mmol) in 30 mL THF. After that, the p‐TsCl solution was dropwise added into the CD solution. The resulting mixture was stirred at room temperature for 4 h and then acetone was introduced at 4 °C to precipitate the product that was subsequently washed twice by diethyl ether, and then recrystallized in H_2_O. The white powder obtained was dissolved in 20 mL DMF to obtain a solution that was added to 7 mL 2,2’‐(ethylenedioxy)bis(ethylamine) (0.048 mol) very slowly under stirring. Then, the reaction mixture was heated to 65 °C and maintained at this temperature for 3 h under nitrogen atmosphere. After the reaction mixture was cooled down to room temperature, the excessive 2,2’‐(ethylenedioxy)bis(ethylamine) was removed by evaporation under reduced pressure. Subsequently, acetone was introduced to precipitate the reaction product that was purified by twice‐recrystallization in H_2_O to obtain the final amino‐modified *β*‐CD (*β*‐CD‐NH_2_).

##### Preparation of Ir‐BTPHSA/CD‐NH_2_ Complex

The Ir‐BTPHSA/CD‐NH_2_ complex complex was prepared by co‐precipitation method. *β*‐CD‐NH_2_ was dissolved in aqueous solution, and Ir‐BTPHSA was dissolved in DMSO. Then the Ir‐BTPHSA solution was slowly dropped into *β*‐CD solution at 1:1 molar ratio of Ir‐BTPHSA to *β*‐CD. The reaction was kept at 45 °C under overnight stirring. Through freeze drying, the final product denoted as Ir‐BTPHSA/CD‐NH_2_ was obtained and subjected to 2D NMR analysis.

##### Synthesis of Ratiometric Fluorescence Oxygen Probe

DMF solution of 1.1 mL containing 0.899 mg (1.1 mmol) of Cy7‐NHS was slowly added into 15 mL deionized water containing 1 mmol Ir‐BTPHSA/CD‐NH_2_. The mixture was heated to 40 °C and maintained at this temperature for 4 h under stirring. After that, the mixture was concentrated with rotary evaporator, followed by introduction of deionized water and low speed centrifugation to remove insoluble impurities. The above purification processes were repeated twice to obtain an aqueous solution of the ratiometric probe denoted as Ir‐BTPHSA/CD‐Cy7 for the following experiments.

##### Cell Hypoxia Imaging In Vitro

Human colorectal cancer cell line LS180 was cultured in high glucose DMEM with 10% fetal bovine serum, 100 U mL^−1^ penicillin, and 0.1 mg mL^−1^ streptomycin at 37 °C under a 5% CO_2_ atmosphere. LS180 cells were first plated into the wells of confocal capsules and incubated overnight at 37 °C under 5% CO_2_ for a firm adherence. Then, the cells were incubated with Ir‐BTPHSA/CD‐Cy7 (0.2 mg mL^−1^) for 2 h at 37 °C. After that, the cells were rinsed twice with PBS to remove the unbound probe for nuclei staining with Hoechst 33 342. Subsequently, the cells obtained were cultured at different O_2_ concentrations maintained by AnaeroPack‐Anaero and AnaeroPack‐Micro Aero for 1 h before further fluorescence imaging with a confocal microscope (Olympus FV 1200). The excitation wavelengths for observing the Ir‐BTPHSA/CD‐Cy7 probe were set at 488 and 647 nm, while the corresponding luminescence windows were set as 550–650 and 700–800 nm, respectively. For nuclei imaging, the excitation line was tuned to 405 nm and the signal window was shifted to 420–470 nm.

##### Animal Tumor Model

The tumor models used were established upon subcutaneous injection of LS180 cells (≈5 × 10^−6^) into male BALB/c nude mice (4–6 weeks old) at the flank region of the right hind leg region. The tumor imaging studies were carried out 7–10 days after the inoculations of tumor cells.

##### In Vivo Hypoxia Imaging

The fluorescence images of a nude mouse bearing a subcutaneous tumor xenograft at the flank region of the right hind leg were acquired with Maestro 2 in vivo spectrum imaging system (Cambridge Research & Instrumentation, Woburn, MA). The tumor‐bearing mice were anesthetized by isoflurane, and then the ratiometric probe was intratumorally injected by a dose level of 20 mg probe per kilogram body weight. For the hypoxia imaging, the excitation light of 465–500 nm was adopted, while the emission window was from 550 to 750 nm. For the fluorescence imaging based on Cy7, a narrow band excitation filter of 640–675 nm was used and the emission range was of 750–950 nm. The exposure time was 300 ms. The imaging results from the chromophore signals were separated through spectral unmixing algorithms with the vendor software.

All animal experiments reported herein were carried out according to a protocol approved by Peking University Institutional Animal Care and Use Committee.

## Conflict of Interest

The authors declare no conflict of interest.

## Supporting information

Supporting Information
