# Supplementary material for: A Cyclodextrin‐Hosted Ir(III) Complex for Ratiometric Mapping of Tumor Hypoxia In Vivo
Source: Adv Sci (Weinh). 2021 Feb 5;8(8):2004044. doi: 10.1002/advs.202004044 (PMC8061356; doi:10.1002/advs.202004044)
Supplement: Supplementary file 1 — Supporting Information [file ADVS-8-2004044-s001.pdf]

WILEY-VCH

© 2020 Wiley-VCH GmbH

ADVANCED SCIENCE
Open Access

Supporting Information

for *Adv. Sci.,* DOI: 10.1002/advs.202004044

A Cyclodextrin-hosted Ir(III) Complex for Ratiometric Mapping of Tumor Hypoxia *in vivo*

Peng Xiao, Chunyan Liu, Tiancong Ma, Xiuhong Lu,
Lihong Jing, Yi Hou, Peisen Zhang, Gang Huang, Mingyuan Gao**

Supporting Information

A Cyclodextrin-hosted Ir(III) Complex for Ratiometric Mapping of Tumor Hypoxia *in vivo*

Peng Xiao, Chunyan Liu, Tiancong Ma, Xiuhong Lu, Lihong Jing, Yi Hou, Peisen Zhang, Gang Huang, Mingyuan Gao**

Key Laboratory of Colloid, Interface and Chemical Thermodynamics, Institute of Chemistry, Chinese Academy of Sciences, Beijing 100190, China; School of Chemistry and Chemical Engineering, University of Chinese Academy of Sciences, Beijing 100049, P. R. China; Shanghai Key Laboratory of Molecular Imaging, Shanghai University of Medicine and Health Sciences, Shanghai 201318, China

Synthesis of $NaGdF_4$:Yb,Tm,Ca@$NaGdF_4$ nanoparticles. Yb,Tm-doped $NaGdF_4$ nanoparticles were prepared according to literature method. Typically, $GdCl_3·6H_2O$ (0.70 mmol), $YbCl_3·6H_2O$ (0.18 mmol), $TmCl_3·6H_2O$ (0.02 mmol), and $CaCl_2$ (0.01mmol) were mixed with 4 mL of OA and 16 mL of ODE in a 100 mL flask. After being heated to 150 °C to form a homogeneous solution under nitrogen protection, the solution was cooled down to 50°C and 10 mL of a methanol solution containing NaOH (1.25 mmol) and NH_4F (2 mmol) was added dropwise. The reaction system was then kept under stirring at 50°C for 30 min. Subsequently, methanol in the system was removed under vacuum at 100°C for 10 min, and the resulting reaction mixture was quickly heated to 300 °C under atmospheric pressure. The reactions were allowed for 1 h under nitrogen protection and then terminated by cooling the reaction mixture to room temperature. The resultant nanoparticles were precipitated by ethanol, collected by centrifugation, washed with ethanol for three times, and finally redispersed in cyclohexane for further experiments.

The following growth of the $NaGdF_4$ shell was carried out by similar procedures for the preparation of $NaGdF_4$: Yb,Tm core particles. Briefly, 9 mL of cyclohexane solution of the purified $NaGdF_4$: Yb,Tm,Ca core nanocrystals was mixed with $GdCl_3·6H_2O$ (0.50 mmol), 4 mL of OA, and 16 mL of ODE in a 100 mL flask. The growth of the $NaGdF_4$ shell and the following purification procedures for the core@shell particles were the same as those for the core nanocrystals. The purified nanoparticles were also redispersed in cyclohexane for further experiments.

Ligand exchange. Approximately 10 mg of the purified particles ($NaGdF_4$:Yb,Tm@$NaGdF_4$) and 100 mg of COOH-PEG-dp were dissolved in 5 mL of THF, and the mixture was kept under stirring overnight at 40°C. After that, the PEGylated particles were precipitated by cyclohexane, washed with cyclohexane for three times, and finally dried under vacuum at room temperature. The obtained nanoparticles were further purified through ultrafiltration with 30 kDa MWCO centrifugal filter (Millipore YM-50) for 4 cycles at 6000 g to remove the free ligand. The nanoparticles finally obtained were characterized by TEM and a representative TEM image is shown in Figure S1.

Synthesis of Ir-BTPHSA-NH_2. Ir-BTPHSA (5.1 mg, 0.007 mmol) and EDC·HCl (2.0 mg, 0.010 mmol) was dissolved in 1 mL CH_2Cl_2. Then 20 μL of 2,2'-(ethylenedioxy)bis(ethylamine) was added into the above solution. The resulting mixture was maintained at room temperature under overnight stirring, and diethyl ether was introduced at 4°C to precipitate the product that was subsequently washed twice by CH_2Cl_2.

Synthesis of Ir-BTPHSA/UCNP conjugate. The Ir-BTPHSA was covalently attached on the surface of the PEGylated UCNPs through the following procedures. Typically, 825 µL of UCNPs aqueous solution (2.1×10^{-7} mol/L) containing 3 mg of EDC·HCl (0.015 mmol) and 4 mg of Sulfo-NHS (0.018 mmol) was prepared and stirred at room temperature for 10 min. Then, 200 µL solution of Ir-BTPHSA in CH_3CN (1.4×10^{-3} mol/L) was added into the above reaction mixture that was then kept at 40 °C under overnight stirring. After that, low speed centrifugation was adopted to remove insoluble impurities, and the resulting solution was subjected to ultrafiltration for 2 cycles with 30 kDa MWCO centrifugal filter to obtain the Ir-BTPHSA/UCNP conjugate.

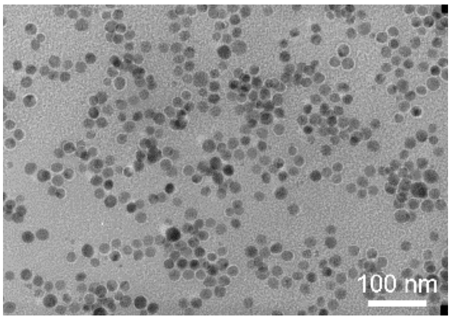

Figure S1. TEM image of the PEGylated $NaGdF_4$:Yb,Tm,Ca@$NaGdF_4$ nanoparticles.

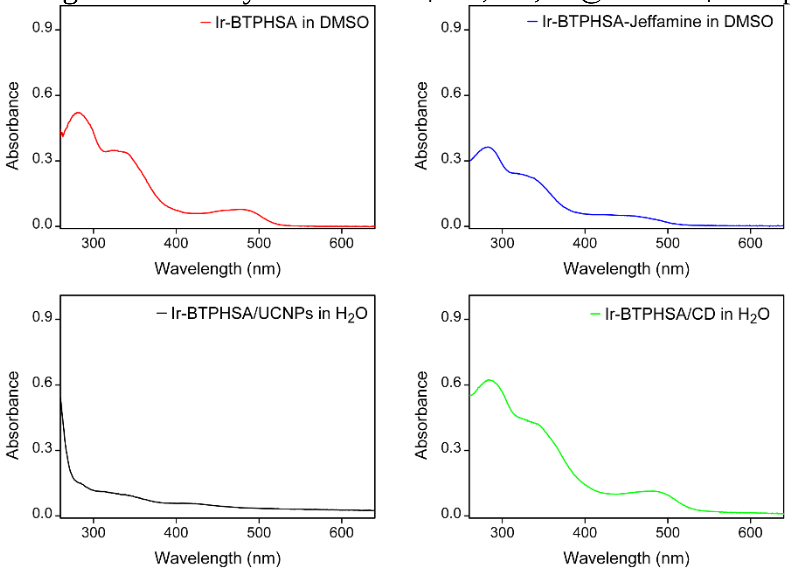

Figure S2. The UV-Vis absorption spectra of Ir-BTPHSA in DMSO, Ir-BTPHSA-jeffamine conjugate in DMSO, Ir-BTPHSA attached on the surface of the PEGylated UCNPs (Ir-BTPHSA/UCNPs) in aqueous solution, and Ir-BTPHSA encapsulated by CDs (Ir-BTPHSA/CD) in aqueous solution.

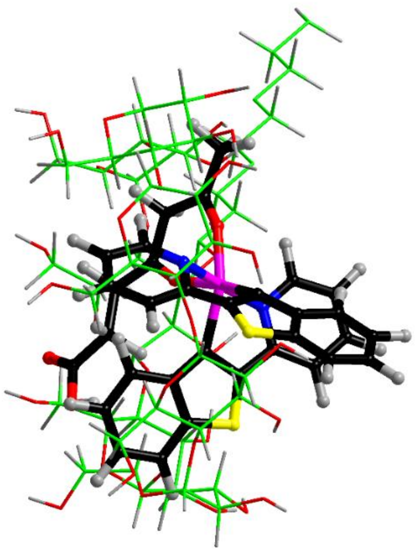

Figure S3. The most possible binding situation between Ir-BTPHSA and β-CD.

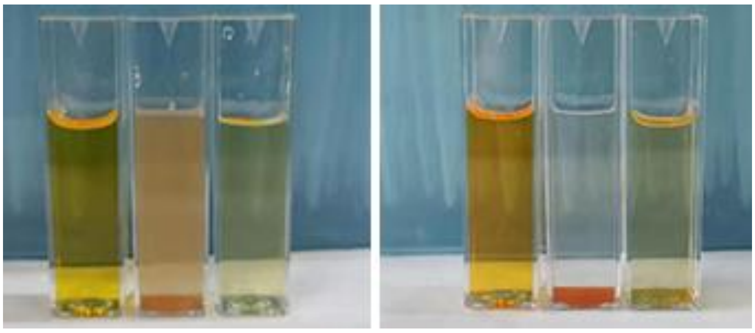

Figure S4. Photographs of Ir-BTPHSA in DMSO (left), Ir-BTPHSA in H_2O (middle), and Ir-BTPHSA/CD in H_2O (right) captured right after the preparation of the solution (left image) and 24 h after the solutions were kept under ambient conditions (right image), respectively.

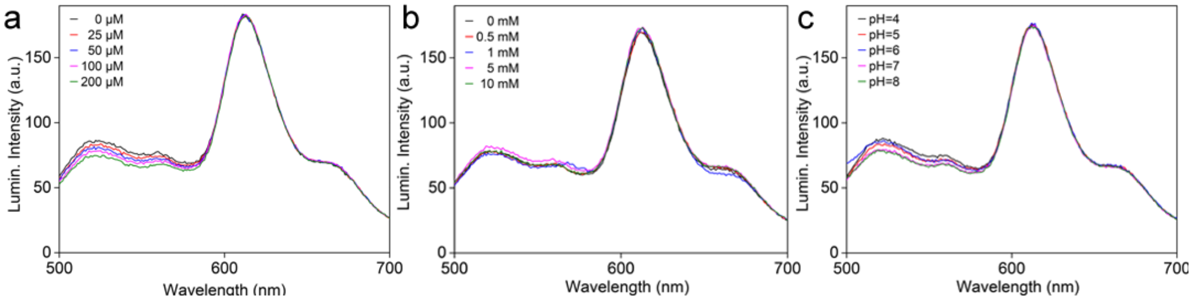

Figure S5. PL spectra of Ir-BTPHSA encapsulated by CDs (ex=488 nm) in aqueous solutions containing different levels H_2O_2 (a), GSH (b), or hydrogen ions (c).

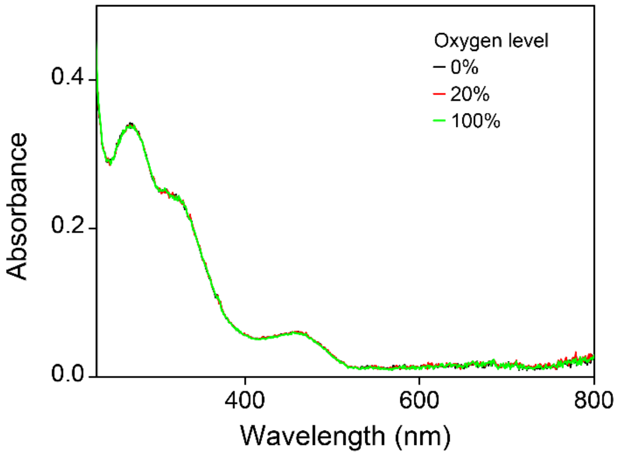

Figure S6. The absorption spectra of Ir-BTPHSA recorded at different the oxygen levels.

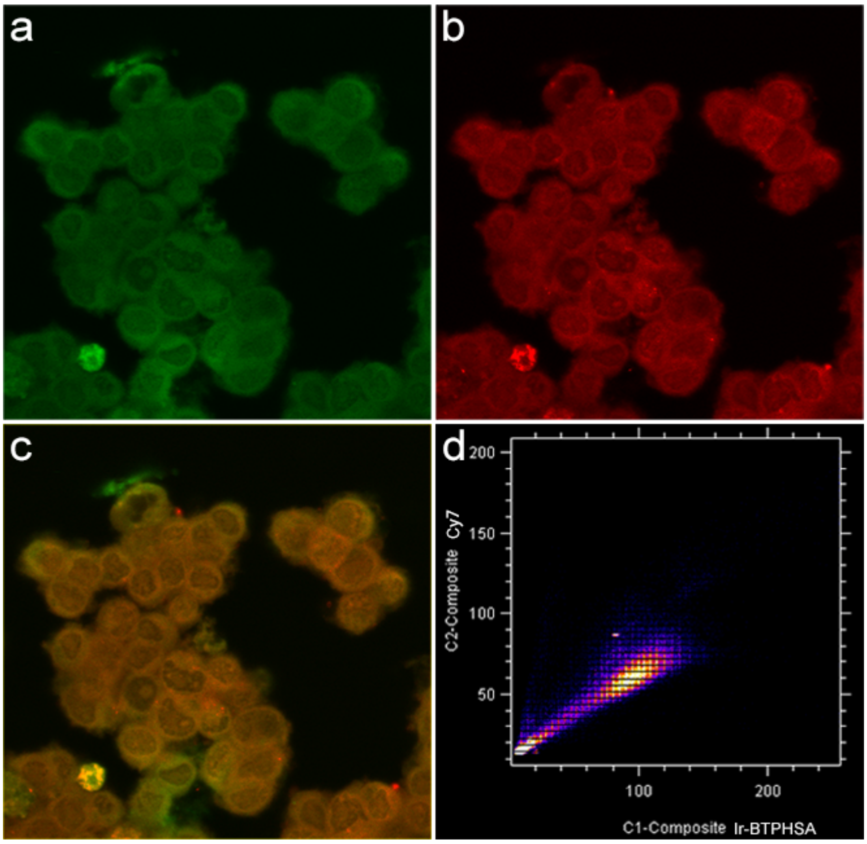

Figure S7. The confocal images of LS180 cells imaged through channels for Ir-BTPHSA (a) and Cy7 (b), respectively, together with the merged image (c) and the intensity correlation plot (d).

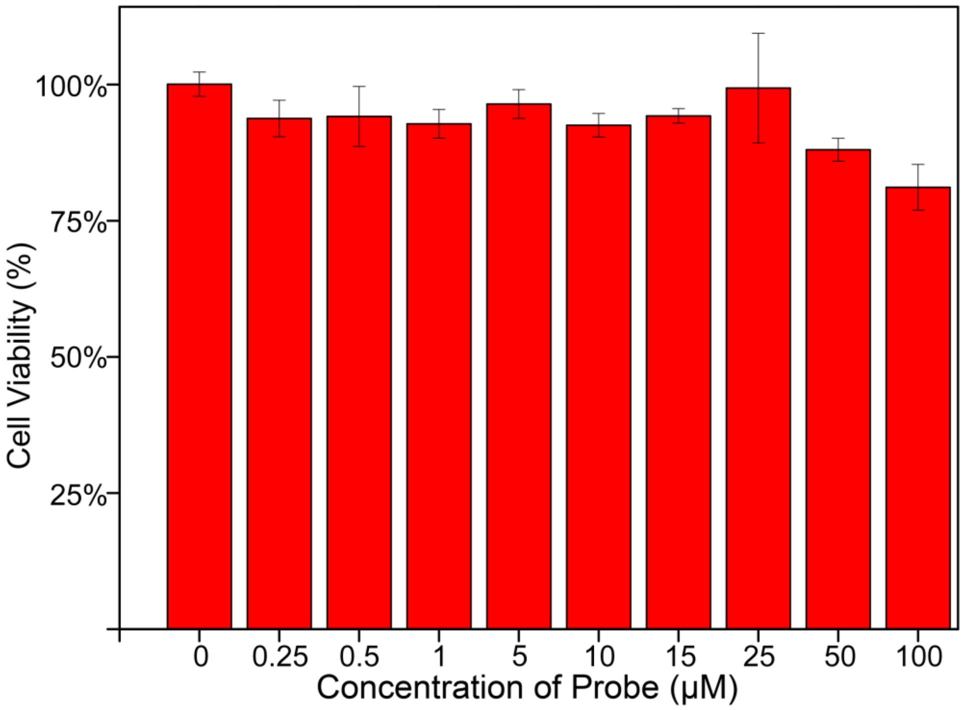

Figure S8. Cytotoxicity test for Ir-BTPHSA/CD-Cy7

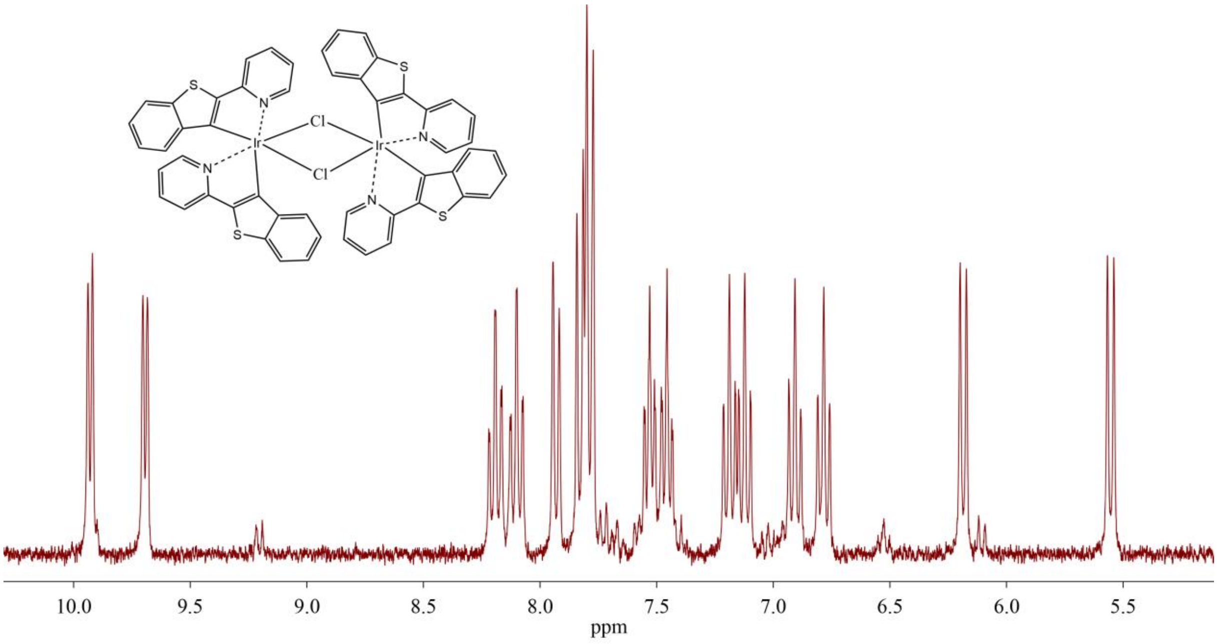

Figure S9. ^{1}H NMR of $[(BTP)_2Ir(\mu\text{-}Cl)]_2$.

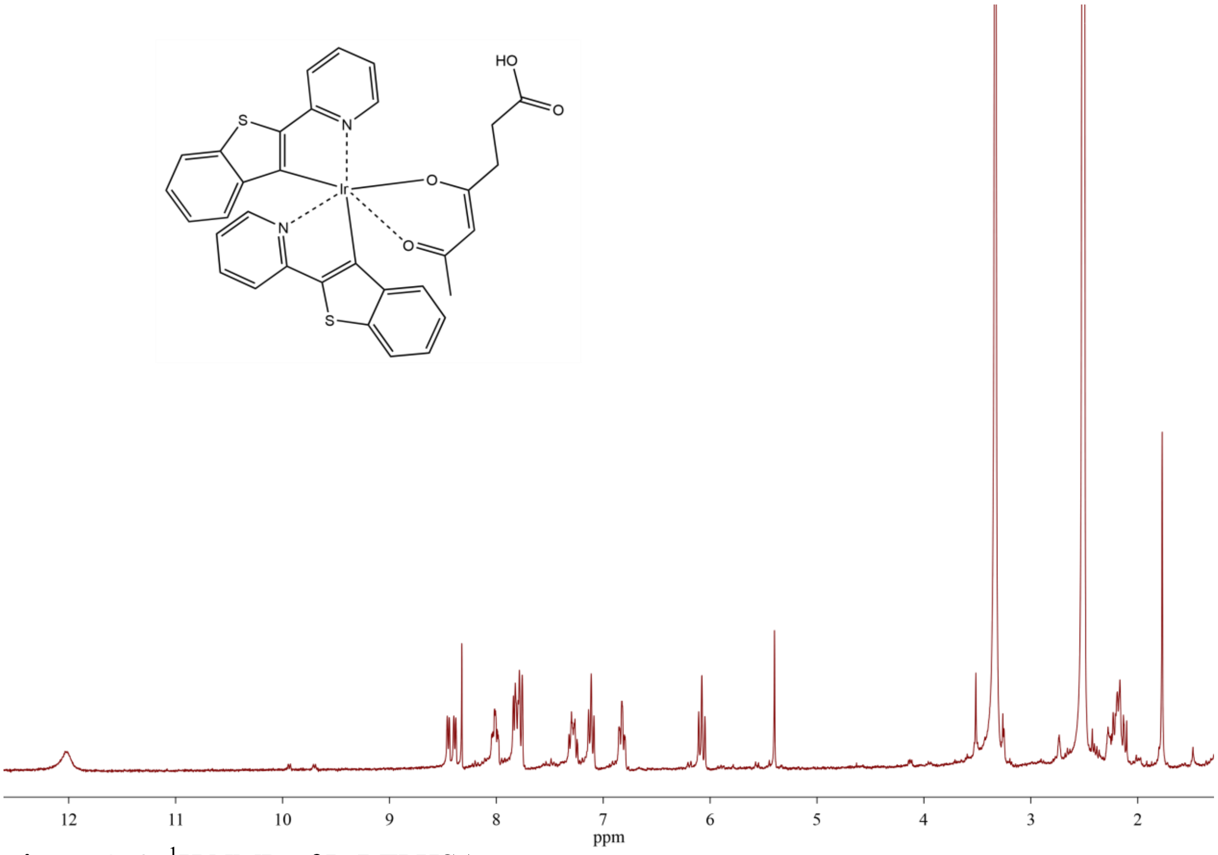

Figure S10. ^{1}H NMR of Ir-BTPHSA.

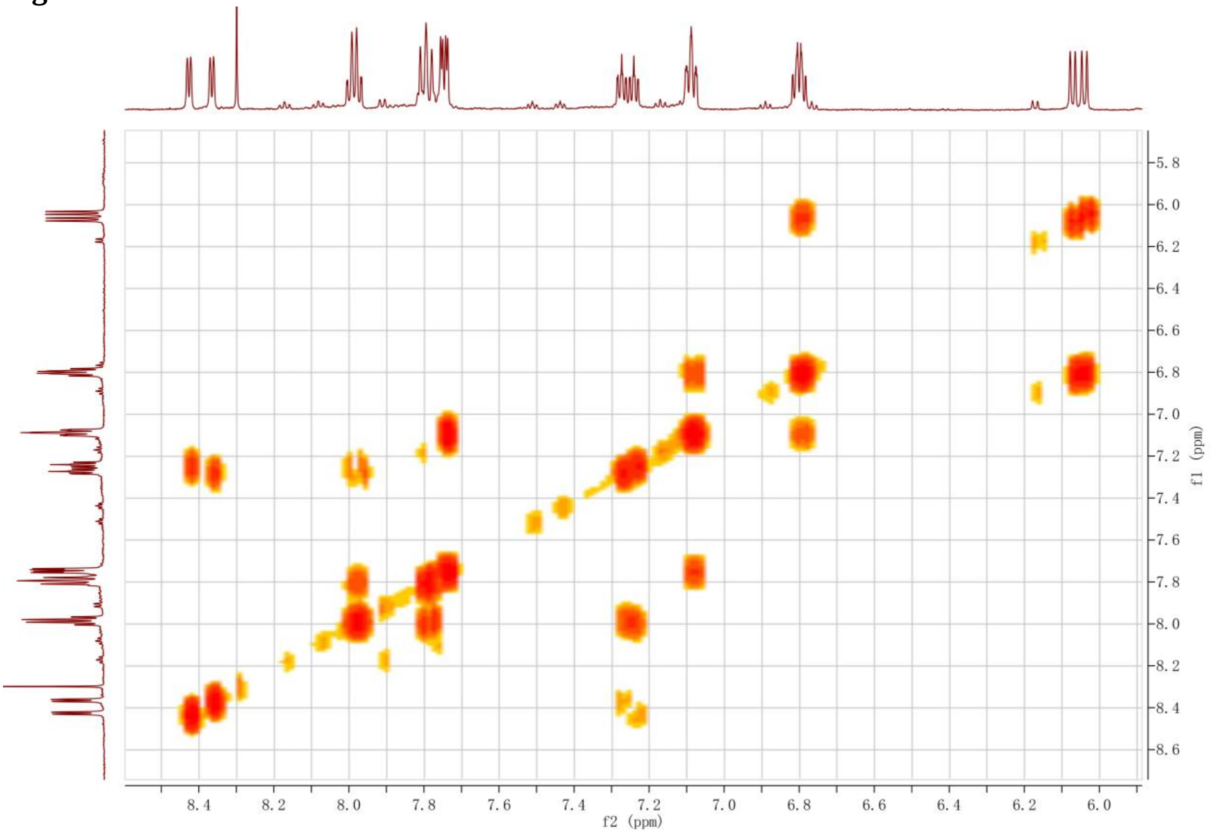

Figure S11. COSY spectra of Ir-BTPHSA
